# Role of age of critically ill children at time of exposure to early or late parenteral nutrition in determining the impact hereof on long-term neurocognitive development: A secondary analysis of the PEPaNIC-RCT

**DOI:** 10.1016/j.clnu.2020.07.004

**Published:** 2021-03

**Authors:** Ines Verlinden, Karolijn Dulfer, Ilse Vanhorebeek, Fabian Güiza, José A. Hordijk, Pieter J. Wouters, Gonzalo Garcia Guerra, Koen F. Joosten, Sascha C. Verbruggen, Greet Van den Berghe

**Affiliations:** a Clinical Division and Laboratory of Intensive Care Medicine, Department of Cellular and Molecular Medicine, KU Leuven, Leuven, Belgium; b Intensive Care Unit, Department of Paediatrics and Paediatric Surgery, Erasmus Medical Centre, Sophia Children's Hospital, Rotterdam, the Netherlands; c Department of Paediatrics, Intensive Care Unit, University of Alberta, Stollery Children's Hospital, Edmonton, Canada

**Keywords:** Pediatric intensive care unit, Critical illness, Parenteral nutrition, Children, Long-term outcome, Neurocognitive development

## Abstract

**Background & aims:**

Early use of parenteral nutrition (early-PN), as compared with withholding it for one week (late-PN), in the PICU, has shown to slow down recovery from critical illness and impair long-term development of 6 neurocognitive/behavioural/emotional functions assessed 2 years later. Given that key steps in brain maturation occur at different times during childhood, we hypothesised that age at time of exposure determines long-term developmental impact of early-PN.

**Methods:**

The 786 children who were neurocognitively tested 2 years after participation in the PEPaNIC-RCT were included in this study. First, for each studied long-term outcome, interaction between randomisation to early-PN versus late-PN and age was assessed with multivariable linear regression analysis. Subsequently, for outcomes with an interaction p ≤ 0.15, the impact of early-PN versus late-PN was analysed, after adjustment for risk factors, for 4 subgroups defined based on developmentally-relevant age at time of exposure [≤28 days (n = 121), 29 days to 11 months (n = 239), 11 months to <5 years (n = 223) and ≥5 years (n = 203)].

**Results:**

Interaction between randomisation and age was present for weight, and parent-reported inhibitory control, cognitive flexibility, working memory, planning/organisation, metacognition, total executive functioning, and internalising and total behavioural/emotional problems. Subgroup analyses revealed that none of the age-groups revealed benefit, whereas children aged 29 days to <11 months were most vulnerable to harm by early-PN for development of inhibitory control (p = 0.008), working memory (p = 0.009), planning/organisation (p = 0.004), metacognition (p = 0.008), and total executive functioning (p = 0.004), and for internalising (p = 0.005) and total behavioural/emotional problems (p = 0.01). Children aged 11 months to <5 years revealed harm by early-PN for development of inhibitory control (p = 0.003). In contrast, children aged ≥5 years and neonates aged ≤28 days appeared less vulnerable.

**Conclusions:**

Critically ill children aged 29 days to 11 months at time of exposure were identified as most vulnerable to developmental harm evoked by early-PN.

**Clinical trials.gov:**

NCT01536275.

## 1 Introduction

Children who have been critically ill often suffer from adverse health sequelae that remain present years after hospital discharge [[1], [2], [3], [4]]. Recently, it has been shown that the nutritional management of patients treated in the paediatric intensive care unit (PICU) can modify short-term outcome as well as the long-term legacy [[5], [6], [7], [8], [9]]. The multicentre ‘Paediatric Early versus Late Parenteral Nutrition in Critical Illness – PEPaNIC’ randomised controlled trial (RCT) has shown that targeting full nutritional intake early by early initiation of supplemental parenteral nutrition when enteral nutrition is insufficient (‘early-PN’) was clinically inferior to accepting the macronutrient deficit that accumulates by postponing any supplemental PN to beyond the first week in the PICU (‘late-PN’) [[6], [7], [8]]. Early-PN was found to increase the risk of infection and to delay recovery from critical illness. Apart from these harmful short-term effects, early-PN also showed to negatively affect the development of 6 neurocognitive and behavioural/emotional functions, as assessed 2 years later, with worse inhibitory control, working memory, metacognition, total executive functioning, more externalising behavioural problems and worse visual-motor integration [9]. These long-term adverse effects of early-PN were found to be mediated by altered DNA-methylation of genes involved in brain development [10].

Given that the age-range of children who are admitted to the PICU is wide (0–17 years old), it is theoretically possible that exposure to early-PN versus late-PN has a different developmental impact depending on the age at time of exposure. Indeed, it is known that exposure to adverse environmental factors during different time windows of childhood can affect brain development either along or away from the normal trajectory [[11], [12], [13]]. Although stages of brain development are not strictly and uniformly timed for an individual child, it is generally accepted that a major brain growth spurt with a steep rise in synaptogenesis for higher cognitive functions occurs from the age of about 1 month until about 11–12 months [11,[14], [15], [16], [17], [18], [19]]. This is followed by a plateau in synaptogenesis and initiation of synapse regression referred to as “pruning” until the age of about 5 years. Thereafter, synaptogenesis tapers off and pruning predominates. Hence, we hypothesised that the age at which children are admitted to the PICU may determine whether exposure to early-PN, as compared with late-PN, evokes long-term developmental harm or benefit. Indeed, although for the total population, early-PN was found to adversely affect development of 6 neurocognitive and behavioural/emotional functions, it is possible that the impact of early PN depends on the age at exposure and, consequently, a neutral outcome for the total patient population may hide benefit for one and harm for another age-group.

To test this hypothesis, we performed a secondary analysis of the PEPaNIC-RCT, in which interaction between randomisation to early-PN versus late-PN and age at time of exposure was first determined for all developmental outcomes assessed at 2-year follow-up, with subsequent subgroup analyses for 4 developmentally-relevant age-groups.

## 2 Materials and methods

### 2.1 Study design and participants

This study is a secondary analysis of the multicentre PEPaNIC-RCT that included 1440 critically ill children (0–17 years) admitted to the PICUs of Leuven (Belgium), Rotterdam (The Netherlands) and Edmonton (Canada) [7]. The full study protocol has been published [6].

**Fig. 1:**
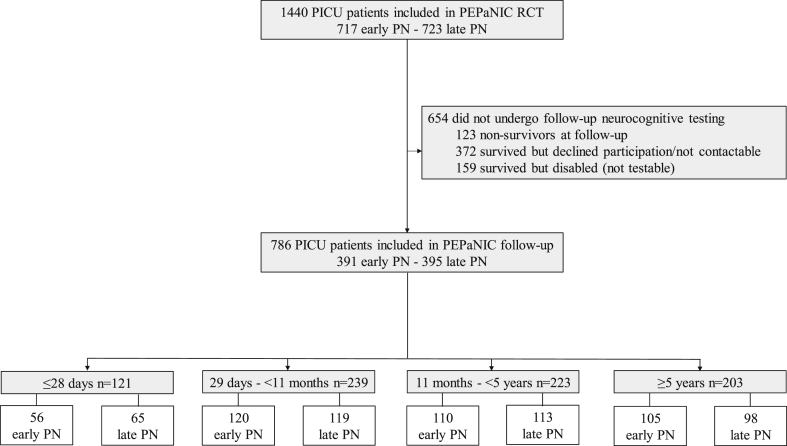
**CONSORT diagram of study participants.** d: days, m: months, PICU: paediatric intensive care unit, PEPaNIC: Paediatric Early versus Late Parenteral Nutrition in Intensive Care Unit, RCT: randomised controlled trial, PN: parenteral nutrition.

From the total PEPaNIC-RCT patient population, 786 patients, 391 from the early-PN group and 395 from the late-PN group, were assessed for physical, neurocognitive and behavioural/emotional functions 2 years later (Fig. 1) [9]. Children who were neonates or infants younger than 6 months old at PICU admission were assessed at the age of 2.5 years, because this is the youngest age for appropriate assessment of parent-reported or caregiver-reported executive functioning (with the Behaviour Rating Inventory of Executive Function [BRIEF]) in combination with a general intelligence test (Wechsler Preschool and Primary Scale of Intelligence [WPPSI]). Inclusion date for follow-up of the other children was 2 years after the date of inclusion in the PEPaNIC-RCT, with an ideal window of 3 months and an accepted window of 6 months before or after this follow-up inclusion date. These 786 patients were included in the present secondary analysis.

Study approval by the institutional review boards of the participating centres (ML8052; NL49708.078; Pro00038098) and written informed consent from the parents or legal guardians and from the child when reaching adolescent age were obtained according to local regulations. The study was performed in accordance with the 1964 Declaration of Helsinki and its amendments.

### 2.2 Randomisation and masking

Patients had been randomised to early-PN or late-PN [6,7]. In the early-PN group, supplemental PN was initiated within 24 h after PICU admission when enteral nutrition was insufficient to reach nutritional targets (Tables S1–2, Fig. S1). In the late-PN group, such supplemental PN was withheld in the first week of PICU stay (which meant no PN for the majority of the patients in view of discharge before day 8) and patients received a mixture of glucose 5% and sodium chloride 0.9% to match fluid intake. After one week, for both groups equally, PN could be administered if necessary. When enteral nutrition covered 80% or more of the calculated targets, supplemental parenteral nutrition was discontinued.

Participants were assessed for developmental outcomes either at the hospital or at home by physicians and experienced paediatric psychologists who were strictly masked for treatment allocation [9]. Parents had not been masked during the time the child was treated in the PICU and were not actively informed about the initial PEPaNIC-study results.

### 2.3 Developmental outcomes

Clinical tests and validated, internationally recognised questionnaires with adequate normative data were used to assess physical, neurocognitive and behavioural/emotional development in the 2-year PEPaNIC follow-up study [1,9]. Studied outcomes available for all ages were growth, performance on clinical neurological examination, executive functions, behavioural and emotional problems, general intellectual functioning and visual-motor integration [9].

To assess growth, body weight, height, and head circumference were measured. A clinical neurological examination was done to assess signs of major neurologic dysfunction in interaction/language skills, gross motor function, involuntary movements, reflexes, coordination and balance, fine motor function, cranial nerves and special senses (sensory, visual and auditory functions). These domains were all scored normal (score 0) or abnormal (score 1), yielding a total score ranging from 0 to 8. Parents or caregivers completed BRIEF questionnaires on executive functioning of their child. For children aged 2.5–5 years 11 months, the preschool version (BRIEF-P) was used and for children aged 6–18 years the child version (BRIEF) was used [20,21]. Only the overlapping domains of the two questionnaires were analysed: inhibitory control, cognitive flexibility, emotional control, working memory, planning and organisation, metacognition and total executive functioning. All scores of the BRIEF questionnaires were reported as T scores, with mean 50 [SD 10]. Higher scores in this questionnaire represent more problems with everyday executive functioning. Parents or caregivers were also asked to complete the Child Behaviour Checklist (CBCL) questionnaires (two versions depending on the age of the child: CBCL 1.5–5 years 11 months or CBCL 6–18 years) to assess behavioural and emotional problems of the children. Internalising, externalising and total behavioural and emotional problems were analysed (T scores with mean 50 [SD 10]) [22,23]. Higher scores on this questionnaire represent more problems. General intellectual ability was assessed with use of the age-appropriate versions of the Wechsler intelligence quotient (IQ) scale. The Wechsler Preschool and Primary Scale of Intelligence WPPSI-III-NL [24] was used for children aged between 2.5 and 5 years 11 months, the Wechsler Intelligence Scale for Children (WISC-III-NL) [25] was used for children aged between 6 and 16 year 11 months, and the Wechsler Adult Intelligence Scale (WAIS-IV-NL) [26] was used for adolescents and young adults who were 17 years or older. For all Wechsler tests, total IQ, verbal IQ, and performance IQ scores (test mean 100 [SD 15]) were computed, with higher scores representing better general intellectual ability. The Beery Developmental Test of Visual-Motor Integration [27] was used to assess the ability to integrate visual and motor functions, involving eye-hand coordination (total scaled score, with test mean 10 [SD 3]). A higher score indicates better visual-motor integration. A detailed description of the outcome measures is available in the appendix.

### 2.4 Statistical analysis

As previously described [9], multiple data imputation by chained equations was performed to correctly address partial responses [28]. To avoid bias and instability in this imputation model, the percentage of missing data per variable could not exceed 30% and thus the number of iterative imputations was set at 31 [9,28].

For each outcome, p-values for the interaction between the randomised intervention (early-PN versus late-PN) and age at time of exposure were determined with use of multivariable linear regression analysis. These analyses were adjusted for the risk factors centre, sex, race, geographical origin, language, the education and occupational status of the parents (appendix), risk of malnutrition (screening tool for risk on nutritional status and growth [STRONGkids] score), severity of illness upon PICU admission (paediatric index of mortality 3 [PIM3] score and paediatric logistic organ dysfunction [PeLOD] score), diagnosis group (surgical-cardiac, surgical-other, neurosurgery/neurology, trauma/burn, transplantation/hematology/oncology, medical-other), history of malignancy, diabetes, a predefined syndrome (appendix), and, parental smoking behaviour. Subsequently, for those outcomes that revealed an interaction p-value ≤0.15, the effect of early-PN versus late-PN was assessed for 4 *a priori* defined age subgroups separately, with multivariable linear regression analysis adjusted for the same risk factors. These 4 *a priori* defined age subgroups [≤28 days old, 29 days to <11 months old, 11 months to <5 years old, and 5 years or older] were identified based on previously reported timing of cerebral maturation spurts and synaptogenesis of higher cognitive functions [11,[14], [15], [16], [17], [18], [19]] and with the aim to obtain, as much as possible, samples of relatively comparable size.

All multivariable linear regression analyses were performed on the 31 imputed datasets with β-estimates and p-values reported as pooled results.

In order to correct for multiple comparisons, two-sided p-values of 0.01 or less were considered statistically significant.

Statistical analyses were performed with use of R version 3.5.3 and JMP© version 14.0.0 (SAS Institute, Inc, Cary, NC).

## 3 Results

Among the 786 children who underwent physical, neurocognitive and behavioural/emotional developmental testing 2 years after randomisation to early-PN or late-PN, 121 were ≤28 days old (56 early-PN and 65 late-PN), 239 were 29 days to <11 months old (120 early-PN and 119 late-PN), 223 were 11 months to <5 years old (110 early-PN and 113 late-PN) and 203 were 5 years or older (105 early-PN and 98 late-PN) (Fig. 1). Patient demographics and medical characteristics upon PICU admission are shown in Table 1. Total energy intake and blood glucose levels of early-PN versus late-PN patients of each age group are shown in Figs. S1–2 for the first 7 days in PICU.

**Table 1:** Patient demographics and medical characteristics.

	≤28 d (n = 121)		29 d - <11 m (n = 239)		11 m - <5 y (n = 223)		≥5 y (n = 203)	
	Early-PN (n = 56)	Late-PN (n = 65)	Early-PN (n = 120)	Late-PN (n = 119)	Early-PN (n = 110)	Late-PN (n = 113)	Early-PN (n = 105)	Late-PN (n = 98)
**Demographics**								
Age								
At randomisation, years	0.02 (0.02)	0.02 (0.02)	0.38 (0.2)	0.37 (0.2)	2.74 (1.1)	2.59 (1.2)	10.24 (3.5)	10.68 (3.7)
At 2-year follow-up, years	2.57 (0.07)	2.56 (0.06)	2.65 (0.2)	2.65 (0.2)	4.61 (1.2)	4.47 (1.2)	12.14 (3.5)	12.55 (3.7)
Sex								
Female	22 (39%)	28 (43%)	42 (35%)	49 (41%)	50 (45%)	50 (44%)	47 (45%)	43 (44%)
Male	34 (61%)	37 (57%)	78 (65%)	70 (59%)	60 (55%)	63 (56%)	58 (55%)	55 (56%)
Known non-white race‡	7 (12%)	2 (3%)	16 (13%)	8 (7%)	7 (6%)	11 (10%)	8 (8%)	4 (4%)
Known non-European origin‡	10 (18%)	8 (12%)	35 (30%)	19 (16%)	22 (20%)	28 (25%)	21 (20%)	9 (9%)
Known non-exclusive Dutch or English language	13 (23%)	11 (17%)	29 (24%)	29 (24%)	24 (22%)	34 (30%)	29 (28%)	15 (15%)
Socioeconomic status								
Parents educational level 1	6 (11%)	8 (12%)	13 (11%)	17 (14%)	10 (9%)	11 (10%)	11 (10%)	15 (15%)
Parents educational level 2	28 (50%)	27 (42%)	46 (38%)	42 (35%)	53 (39%)	52 (46%)	39 (37%)	38 (38%)
Parents educational level 3	11 (20%)	20 (31%)	33 (28%)	37 (31%)	32 (29%)	19 (17%)	24 (23%)	24 (23%)
Parents educational level unknown	11 (20%)	10 (15%)	28 (23%)	23 (19%)	25 (23%)	31 (27%)	31 (30%)	21 (21%)
Parents occupational level 1	6 (11%)	8 (12%)	11 (9%)	14 (12%)	6 (5%)	16 (14%)	12 (11%)	13 (13%)
Parents occupational level 2	10 (18%)	16 (25%)	32 (27%)	31 (26%)	30 (27%)	16 (14%)	33 (31%)	24 (24%)
Parents occupational level 3	11 (20%)	14 (22%)	29 (24%)	25 (21%)	29 (26%)	28 (25%)	17 (16%)	26 (27%)
Parents occupational level 4	7 (13%)	13 (20%)	16 (13%)	25 (21%)	17 (15%)	24 (22%)	13 (12%)	5 (5%)
Parents occupational level unknown	22 (39%)	14 (22%)	32 (27%)	24 (20%)	28 (25%)	33 (29%)	30 (29%)	30 (31%)
**Patient characteristics upon PICU admission**								
STRONGkids risk level								
Medium	39 (70%)	54 (83%)	109 (91%)	105 (88%)	106 (96%)	105 (93%)	97 (92%)	92 (94%)
High	17 (30%)	11 (17%)	11 (9%)	14 (12%)	4 (4%)	8 (7%)	8 (8%)	6 (6%)
PeLOD score. first 24 h in PICU	17.8 (11.7)	17.9 (11.0)	20.7 (11.2)	21.9 (11.5)	21.7 (11.8)	19.8 (11.1)	18.5 (11.7)	19.5 (12.1)
PIM3 score	−2.87 (1.3)	−3.1 (1.4)	−3.6 (1.3)	−3.5 (1.2)	−3.4 (1.4)	−3.6 (1.3)	−3.6 (1.5)	−3.7 (1.5)
Diagnostic category								
Surgical-cardiac	20 (36%)	23 (35%)	70 (58%)	60 (50%)	47 (43%)	46 (41%)	36 (34%)	37 (38%)
Surgical-other	25 (45%)	28 (43%)	16 (13%)	17 (14%)	13 (12%)	12 (11%)	13 (12%)	15 (15%)
Neurosurgery/neurology	0 (0%)	0 (0%)	10 (8%)	12 (10%)	21 (19%)	21 (19%)	29 (28%)	22 (22%)
Trauma/burn	0 (0%)	0 (0%)	0 (0%)	0 (0%)	5 (5%)	3 (3%)	10 (10%)	7 (7%)
Transplantation/hematology/oncology	0 (0%)	0 (0%)	1 (0.8%)	0 (0%)	2 (2%)	8 (7%)	6 (6%)	5 (5%)
Medical-other	11 (20%)	14 (22%)	23 (19%)	30 (25%)	22 (20%)	23 (20%)	11 (10%)	12 (12%)
History of malignancy	0 (0%)	0 (0%)	1 (0.08%)	0 (0%)	10 (9%)	7 (6%)	15 (14%)	9 (9%)
Diabetes	0 (0%)	0 (0%)	0 (0%)	0 (0%)	0 (0%)	0 (0%)	1 (1%)	0 (0%)
Predefined syndrome	3 (5%)	5 (8%)	10 (8%)	16 (13%)	12 (11%)	14 (12%)	9 (9%)	10 (10%)
Parental smoking between birth and PICU admission	26 (48%)	22 (34%)	47 (39%)	40 (34%)	54 (49%)	61 (54%)	57 (54%)	47 (48%)

Data are mean (SD) or n (%). STRONGkids: screening tool for risk on nutritional status and growth. PeLOD: paediatric logistic organ dysfunction. PIM3: paediatric index of mortality 3. PICU: paediatric intensive care unit. ‡ Participants were classified according to race and geographical origin by the investigators. The educational and occupational level is the mean of the paternal and maternal educational or occupational level (appendix). STRONGkids scores range from 0 to 5, with a score of 0 indicating a low risk of malnutrition, a score of 1–3 indicating a medium risk, and a score of 4–5 indicating a high risk. PeLOD scores range from 0 to 71, with higher scores indicating more severe illness. Higher PIM3 scores indicate a higher risk of mortality. ‘Surgical-other’ includes abdominal, thoracic or, other surgery. ‘Medical-other’ includes cardiac, gastrointestinal or hepatic, renal, respiratory, or other medical problems. A predefined syndrome is any pre-randomisation syndrome or illness *a priori* defined as affecting or possibly affecting neurocognitive development (appendix).

Interaction between randomisation to early-PN versus late-PN and age at time of exposure was identified for 9 developmental outcomes: weight, development of inhibitory control, cognitive flexibility, working memory, planning and organisation, metacognition, and total executive functioning, and internalising and total behavioural and emotional problems (Table 2). No interaction between randomisation to early-PN versus late-PN and age at exposure was present for height, head circumference, clinical neurological evaluation score, externalising behavioural problems, verbal IQ, performance IQ and total IQ and visual-motor-integration. Hence, the harmful effect of randomisation to early-PN, versus late-PN, previously identified for externalising behavioural and emotional problems and visual-motor-integration, was not determined by age at time of exposure and was present across all ages [9].

**Table 2:** Interaction between randomisation to early-PN or late-PN and age at time of exposure in determining developmental outcome (physical, neurocognitive and behavioural/emotional functions) two years later.

Developmental outcome	Interaction p-value
Physical development	
Weight	0.01∗
Height	0.93
Head circumference	0.28
Clinical neurological evaluation score	0.86
Parent- or caregiver-reported executive functions	
Inhibitory control	0.10∗
Cognitive flexibility	0.15∗
Emotional control	0.29
Working memory	0.02∗
Planning and organisation	0.004∗
Metacognition	0.01∗
Total executive functioning	0.01∗
Parent- or caregiver-reported behavioural/emotional problems	
Internalising behavioural/emotional problems	0.15∗
Externalising behavioural/emotional problems	0.21
Total behavioural and emotional problems	0.07∗
IQ	
Verbal IQ	0.99
Performance IQ	0.60
Total IQ	0.72
Visual-motor integration	0.40

For each developmental outcome, p-values for interaction between randomisation to early-PN or late-PN and age at randomisation were determined with multivariable linear regression analyses in which age was entered as a continuous variable. ∗interaction p-value ≤0.15. Results are computed from the 31 datasets generated by multiple data imputation by chained equations under a missing-at-random assumption. Covariates entered in the multivariable analyses are: centre, sex, race, geographical origin, language, the education and occupational status of the parents (appendix), risk of malnutrition (screening tool for risk on nutritional status and growth [STRONGkids] score), severity of illness upon PICU admission (paediatric index of mortality 3 [PIM3] score and paediatric logistic organ dysfunction [PeLOD] score), diagnosis group (surgical-cardiac, surgical-other, neurosurgery/neurology, trauma/burn, transplantation/hematology/oncology, medical-other), history of malignancy, diabetes, a predefined syndrome (appendix), and, parental smoking behaviour before PICU admission.

**Fig. 2:**
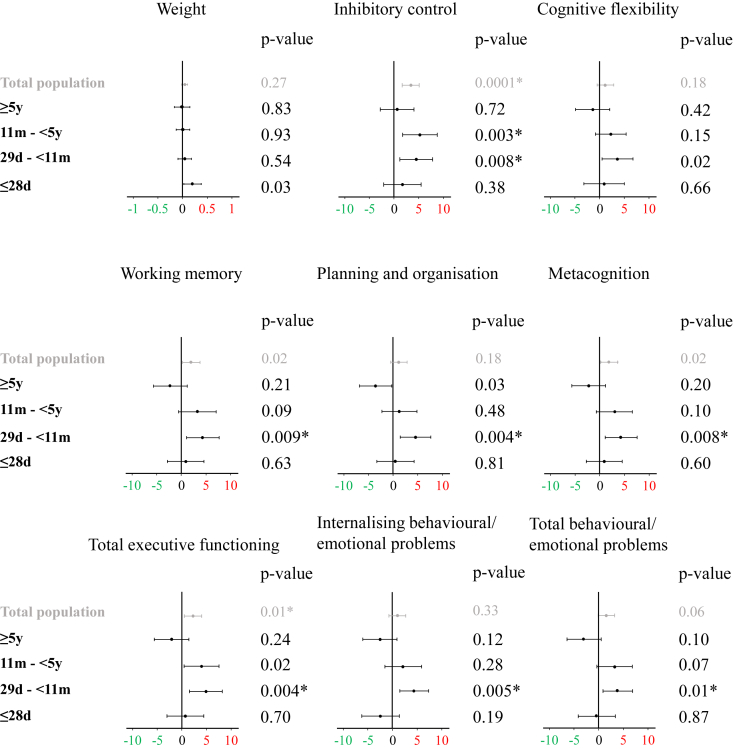
**Impact of early-PN, as compared with late-PN, during stay in the PICU on developmental outcome (physical, neurocognitive and behavioural/emotional functions) of children assessed two years later, according to age at time of exposure.** Data are presented as β-estimate (dot) and 95% CI (line) for the effect of early-PN, versus late-PN, on those outcomes that revealed interaction between randomization and age at time of exposure. Results represent the combined β-estimates and p-values from 31 datasets generated by multiple data imputation by chained equations under a missing-at-random assumption for 786 PEPaNIC patients divided into 4 *a priori* defined developmentally-relevant age categories, with data from the total study population, previously published [9], added for comparison. β-estimates were adjusted for centre, sex, race, geographical origin, language, the education and occupational status of the parents (appendix), risk of malnutrition (screening tool for risk on nutritional status and growth [STRONGkids] score), severity of illness upon PICU admission (paediatric index of mortality 3 [PIM3] score and paediatric logistic organ dysfunction [PeLOD] score), diagnosis group (surgical-cardiac, surgical-other, neurosurgery/neurology, trauma/burn, transplantation/hematology/oncology, medical-other), history of malignancy, diabetes, a predefined syndrome (appendix), and parental smoking behaviour. For the developmental outcomes red indicates worse scores and green indicates better scores. For weight, red indicates ‘heavier’ and green indicates ‘lighter’ weight-for-age z-score than the average for that age. (For interpretation of the references to color in this figure legend, the reader is referred to the Web version of this article.)

For the 9 outcomes that revealed interaction between randomisation to early-PN versus late-PN and age at time of exposure, none of the age subgroups showed benefit from early-PN. Instead, interaction between randomisation to early-PN versus late-PN and age at time of exposure revealed that one subgroup was particularly vulnerable to harm evoked by early-PN whereas other subgroups were less vulnerable. More specifically, neonates aged ≤28 days old and children aged 5 years or older at time of exposure did not appear to suffer from harm by early-PN (Fig. 2). In contrast, for children aged between 29 days and 11 months at time of exposure, patients in the early-PN group performed much worse than those in the late-PN group for most neurocognitive and behavioural/emotional functions that showed interaction with age, but not for weight (Fig. 2). More specifically, children aged between 29 and 11 months at time of exposure to early-PN had worse inhibitory control (β-estimate 4.54, 95% CI 1.21 to 7.87; p = 0.008), working memory (β-estimate 4.35, 95% CI 1.10 to 7.60; p = 0.009), planning and organisation (β-estimate 4.49, 95% CI 1.41 to 7.57; p = 0.004), metacognition (β-estimate 4.42, 95% CI 1.15 to 7.69; p = 0.008) and overall executive functioning (β-estimate 4.84, 95% CI 1.55 to 8.13; p = 0.004) than children exposed to late-PN. Parents or caregivers also reported more behavioural and emotional problems for children aged between 29 and 11 months at time of exposure to early-PN, as compared with late-PN, with more internalising problems (β-estimate 4.23, 95% CI 1.31 to 7.15; p = 0.005) and total behavioural and emotional problems (β-estimate 3.98, 95% CI 0.96 to 7; p = 0.01) (Fig. 2). For children aged between 11 months and 5 years at time of exposure, lower scores were observed with early-PN, as compared with late-PN, for inhibitory control (β-estimate 5.29, 95% CI 1.78 to 8.80; p = 0.003), but not for any of the other outcomes (Fig. 2).

## 4 Discussion

This secondary analysis of the PEPaNIC-RCT and its 2-year follow-up study revealed that the administration of early-PN as compared with omitting PN for one week had a different impact on 9 long-term developmental outcomes depending on the age at time of exposure. No age group revealed long-term developmental benefit from early-PN, whereas children aged between 29 days and 11 months at time of exposure, as compared with other age subgroups, appeared most vulnerable to the long-term developmental harm evoked by early-PN.

This secondary analysis of the 2-year follow-up of the PEPaNIC-RCT revealed that there was interaction between randomisation to early-PN versus late-PN and age at time of exposure in determining the long-term developmental consequences of the use of early-PN. Unlike what we had hypothesised, there was no specific age subgroup that benefited from the use of early-PN that could have been hidden by a neutral outcome in the total PEPaNIC population [9]. Even if not taking into account correction for multiple comparisons, early-PN appeared to affect only a single outcome, i.e. development of planning and organisation, in a positive manner. Instead, the interaction between randomisation to early-PN versus late-PN and age at time of exposure pointed towards a specific age subgroup that was particularly vulnerable to the long-term developmental harm induced by early-PN, whereas this was less so for the other age subgroups. Indeed, critically ill children exposed to early-PN at an age between 29 and 11 months suffered most, whereas patients who were neonates, aged ≤28 days, and older children aged 5 years or more at time of exposure were least harmed by the use of early-PN, except for the two outcomes previously found to be negatively affected by early-PN in the total population - externalising behavioural and emotional problems and visual-motor-integration - but for which there was no interaction with age at exposure. Children aged between 29 days and 11 months at time of exposure to early-PN, versus late-PN, had worse scores for the development of 5 higher cognitive functions (inhibitory control, working memory, metacognition, planning and organisation, total executive functioning) and suffered more from internalising and total behavioural and emotional problems as compared with the other age subgroups. The adverse effect of early-PN on development of planning and organisation, internalising and total behavioural and emotional problems documented in this age-group, had not been identified earlier for the total PEPaNIC study population. For development of working memory, metacognition and total executive functioning, the adverse effect of early-PN was larger in this age subgroup than previously identified for the total patient population [9]. Although children aged between 11 months and 5 years at time of exposure to early-PN also suffered from impaired development of inhibitory control, other developmental outcomes were unaffected. Interestingly, both the critically ill term neonates (aged ≤28 days) and the oldest children aged 5 years or more appeared least vulnerable to long-term harm evoked by early-PN during critical illness.

Our finding that patients aged between 29 days and 11 months were most vulnerable to adverse long-term effects of exposure to early-PN in the PICU was in line with the first year of life being critical for brain development and with the known high sensitivity of the brain to environmental disturbances during this time window [[11], [12], [13], [14], [15], [16], [17], [18], [19]]. Indeed, postnatally, a brain growth spurt takes place roughly between 1 and 11 months of postnatal age, a time window during which many “sensitive” and “critical” periods have been identified. Various potentially harmful environmental exposures such as psychological stressors (caregiver insensitivity, violence), malnutrition (under- and overfeeding) and infectious and noninfectious inflammation can have major impact on brain growth and maturation whereby they can affect long-term development leading to increased risk of cognitive, emotional and social deficits [2,11,[29], [30], [31]]. Also children aged between 11 months and 5 years showed early-PN induced harm in particular for the development of inhibitory control, which may point to an effect on the initiation of the pruning process which is important for normal development of executive functioning [11,16,17,30].

Our observation that children older than 5 years seemed less vulnerable to the cognitive harm evoked by early-PN during critical illness is in line with the general knowledge that beyond this age, fewer “critical” and “sensitive” windows of brain development occur [[11], [12], [13], [14], [15], [16], [17], [18], [19],^30^,^31^]. Less expected was the observation that patients who were at neonatal age at time of exposure to early-PN in the PICU did not show neurocognitive developmental harm evoked by early-PN except for an increased risk of impaired visual motor integration and disturbed externalising behaviour [9] which did not depend on age of exposure as shown here. This is in contrast with the particularly high vulnerability to the short-term harm evoked by early-PN in this age group, as reported previously [8]. One could speculate about possible explanations. First, during the initial 4 weeks of postnatal life, predominantly sensory functions rather than higher cognitive functions are being developed [11,16,17,30]. Second, in the context of brain damage, it has been suggested that the younger the patient at the time of the insult, the better the recovery [32]. However, specific studies that focus on term neonates are currently lacking. Our finding of protection against harm from early-PN in this youngest age-group may be explained either by a predominant adverse effect of early-PN on synaptogenesis for higher cognitive functions, or it may suggest that neonates can better overcome such a metabolic insult.

This study has some limitations to highlight. By dividing the PEPaNIC patients into developmentally-relevant age subgroups, statistical power was inevitably reduced as compared with the original patient population. However, we based our conclusion on age-dependent vulnerability to harm evoked by early-PN via assessing, in the total patient population, the statistical interaction between randomisation to early-PN versus late-PN and age at time of exposure, which circumvented such a power issue. Hypothesis-generating, the visualisation of the differences in impact of early-PN versus late-PN per *a priori* defined age subgroup clearly identified one particularly vulnerable age subgroup, despite its smaller sample size. A second limitation is the fact that the sample size of the subgroup of neonates at time of exposure was somewhat smaller than that of the other subgroups. This may have reduced the statistical power to detect vulnerability in this youngest subgroup. However, most of the confidence intervals for the effect of early-PN versus late-PN were symmetrically spanning neutrality. Finally, energy requirements were mostly estimated by standard equations rather than by indirect calorimetry, which has been criticised for risk of overfeeding. However, macronutrient doses administered to patients in the early-PN group were substantially below target [33]. Furthermore, also the use of indirect calorimetry for estimating energy expenditure in critically ill children has been criticised for accuracy [34] and feasibility [35], and hence is not frequently used in daily practice [36]. Nevertheless, in hindsight, the children treated with early-PN can be considered overfed in view of the adverse outcomes reported in this group, even with low doses [[7], [8], [9]].

In conclusion, the negative impact of early-PN in critically ill children on development of visual-motor-integration and externalising behaviour, assessed 2 years later, was present across all ages. We could not identify an age subgroup of patients that benefited from early-PN for the long-term physical and neurocognitive development. In contrast, in particular critically ill children aged between 29 days and 11 months at time of exposure to early-PN were identified as most vulnerable to the long-term developmental harm evoked by early-PN. These findings further support de-implementation of the use of early-PN in critically ill children of all ages.

## Statement of authorship

IV, KD, IVH, FG and GVdB designed the study: conceptualisation, methodology. KD, JAH, PJW GGG, KJ and SCV gathered data: resources, data curation. IV, KD, IVH, FG, and GVdB analysed the data and wrote the manuscript: formal analysis, investigation, visualisation, writing – original draft; which was reviewed and approved by all authors: writing – review & editing. GGG, KJ, SCV and GVdB supervised the project in the respective centres: supervision, project administration, funding acquisition. All authors jointly decided to publish. GVdB had full access to all the data in the study and takes responsibility for the integrity of the data and the accuracy of the data analysis.

## Funding sources

This work was supported by an ERC Advanced Grant (AdvG-2012-321670) from the Ideas Program of the EU FP7 and by an ERC Advanced Grant (AdvG-2017-785809) from the Horizon 2020 Program of the EU to GVdB by the Methusalem program of the Flemish government (through the University of Leuven to GVdB, METH/08/07 and to GVdB and IV, METH14/06); by the Institute for Science and Technology, Flanders, Belgium (through the University of Leuven to GVdB, IWT/070695/TBM); by the Sophia Foundation (SSWO) to SV; by the Stichting Agis Zorginnovatie to SV; Nutricia Research B.V to SV; by the Erasmus Trustfonds to SV; and by an European Society for Clinical Nutrition and Metabolism (ESPEN) research grant to SV.

## Role of funding sources

The funders of the study had no role in study design, data collection, data analysis, data interpretation, writing of the report, or the decision to submit for publication.

## Conflict of Interest

We declare no competing interests.

## References

[1] Mesotten D., Gielen M., Sterken C., Claessens K., Hermans G., Vlasselaers D. (2012). Neurocognitive development of children 4 years after critical illness and treatment with tight glucose control: a randomized controlled trial. J Am Med Assoc.

[2] Kachmar A.G., Irving S.Y., Connolly C.A., Curley M.A.Q. (2018). A systematic review of risk factors associated with cognitive impairment after pediatric critical illness. Pediatr Crit Care Med.

[3] Schiller R., IJsselstijn H., Hoskote A., White T., Verhulst F., van Heijst A. (2018). Memory deficits following neonatal critical illness: a common neurodevelopmental pathway. Lancet Child Adolesc Health.

[4] Verstraete S., Van den Berghe G., Vanhorebeek I. (2018). What's new in the long-term neurodevelopmental outcome of critically ill children. Intensive Care Med.

[5] Mehta N.M., Bechard L.J., Cahill N., Wang M., Day A., Duggan C.P. (2012). Nutritional practices and their relationship to clinical outcomes in critically ill children - an international multicenter cohort study. Crit Care Med.

[6] Fivez T., Kerklaan D., Verbruggen S., Vanhorebeek I., Verstraete S., Tibboel D. (2015). Impact of withholding early parenteral nutrition completing enteral nutrition in pediatric critically ill patients (PEPaNIC trial): study protocol for a randomized controlled trial. Trials.

[7] Fivez T., Kerklaan D., Mesotten D., Verbruggen S., Wouters P.J., Vanhorebeek I. (2016). Early versus late parenteral nutrition in critically ill children. N Engl J Med.

[8] van Puffelen E., Vanhorebeek I., Joosten K.F.M., Wouters P.J., Van den Berghe G., Verbruggen S.C.A.T. (2018). Early versus late parenteral nutrition in critically ill term neonates: a preplanned secondary subgroup analysis of the PEPaNIC multicentre randomised controlled trial. Lancet Child Adolesc Health.

[9] Verstraete S., Verbruggen S.C., Hordijk J.A., Vanhorebeek I., Dulfer K., Güiza F. (2019). Long-term developmental effects of withholding parenteral nutrition for 1 week in the paediatric intensive care unit: a 2-year follow-up of the PEPaNIC international randomised controlled trial. Lancet Respir Med.

[10] Guïza F., Vanhorebeek I., Verstraete S., Verlinden I., Derese I., Ingels C. (2020). Effect of early parenteral nutrition during paediatric critical illness on dna methylation as a potential mediator of impaired neurocognitive developement: a pre-planned secondary analysis of the PEPaNIC international randomised controlled trial. Lancet respir med.

[11] Bhutta Z.A., Guerrant R.L., Nelson C.A. (2017). Neurodevelopment, nutrition and inflammation: the evolving global child health landscape. Pediatrics.

[12] Fox S.E., Levitt P., Nelson C.A. (2010). How the timing and quality of early experiences influence the development of brain architecture. Child Dev.

[13] Bruer J.T. (2001). Critical and sensitive period primer. Paul H. Critical thinking about critical periods.

[14] Kolb B., Fantie D. (2009). Development of the child's brain and behavior. Reynolds CR, Fletcher-Janzen E: handbook of clinical child neuropsychology.

[15] Epstein H.T. (1978). Growth spurts during brain development: implications for educational policy and practice. Chall JS,Mirsky AF: education and the brain.

[16] Huttenlocher P.R. (1979). Synaptic density in human frontal cortex - developmental changes and effects of aging. Brain Res.

[17] Thompson R.A., Nelson C.A. (2001). Developmental science and the media. Early brain development. Am Psychol.

[18] Knickmeyer R.C., Gouttard S., Kang C., Evans D., Wilber K., Smith J.K. (2008). Structural MRI study of human brain development from birth to 2 years. J Neurosci.

[19] Chugani H.T. (2018). Imaging brain metabolism in the newborn. J Child Neurol.

[20] Van der Heijden K.B., Suurland J., De Sonneville L.M., Swaab H. (2013). BRIEF-P Vragenlijst voor executieve functies voor 2- tot 5-jarigen: Handleiding.

[21] Huizinga M., Smidts D. (2012). BRIEF Vragenlijst executieve functies voor 5- tot 18-jarigen: Handleiding.

[22] Achenbach T.M., Rescorla L.A. (2000). Manual for the ASEBA preschool forms and profiles.

[23] Verhulst F.C., Van der Ende J., Handleiding A.S.E.B.A. (2013). Vragenlijsten voor leeftijden 6 tot en met 18 jaar [ASEBA Manual Questionnaires for ages 6 to 18 years].

[24] Hendriksen J., Hurks P., WPPSI N.L. (2010). Wechsler preschool and primary Scale of intelligence: handleiding.

[25] Wechsler D. (2005). WISC-III Nederlandstalige bewerking. Handleiding.

[26] Wechsler D. (2012). WAIS-III Nederlandstalige bewerking. Afname en Scoringshandleiding.

[27] Beery K.E., Buktenica N.A., Beery N.A. (2010). The beery-buktenica developmental test of visual-motor integration.

[28] Wulff J., Jeppesen L. (2017). Multiple imputation by chained equations in praxis: guidelines and review. Electron J Bus Res Methods.

[29] Max J.E., Bruce M., Keatley E., Delis D. (2010). Pediatric stroke: plasticity, vulnerability, and age of lesion onset. J Neuropsychiatry Clin Neurosci.

[30] Nelson C.A., Zeanah C.H., Fox N.A. (2019). How early experience shapes human development: the case of psychosocial deprivation. Neural Plast.

[31] Tierney A.L., Nelson C.A. (2009). Brain development and the role of experience in the early years. Zero Three.

[32] Webb C., Rose F.D., Johnson D.A., Attree E.A. (1996). Age and recovery from brain injury: clinical opinions and experimental evidence. Brain Inj.

[33] Vanhorebeek I., Verbruggen S., Casaer M.P., Gunst J., Wouters P.J., Hanot J. (2017). Effect of early supplemental parenteral nutrition in the paediatric ICU: a preplanned observational study of post-randomisation treatments in the PEPaNIC trial. Lancet Respir Med.

[34] Larsen B.M.K., Beggs M.R., Leong A.Y., Kang S.H., Persad R., Garcia Guerra G. (2018). Can energy intake alter clinical and hospital outcomes in PICU?. Clin Nutr ESPEN.

[35] Beggs M.R., Garcia Guerra G., Larsen B.M.K. (2016). Do PICU patients meet technical criteria for performing indirect calorimetry*?*. Clin Nutr ESPEN.

[36] Kerklaan D., Fivez T., Mehta N.M., Mesotten D., van Rosmalen J., Hulst J.M. (2016). Worldwide survey of nutritional practices in PICUs. Pediatr Crit Care Med.

