# Supplementary material for: Role of age of critically ill children at time of exposure to early or late parenteral nutrition in determining the impact hereof on long-term neurocognitive development: A secondary analysis of the PEPaNIC-RCT
Source: Clin Nutr. 2021 Mar;40(3):1005–12. doi: 10.1016/j.clnu.2020.07.004 (PMC7957365; doi:10.1016/j.clnu.2020.07.004)
Supplement: Multimedia component 1 [file mmc1.docx]

**SUPPLEMENTARY MATERIAL**

**Supplement to:**

**Role of age of critically ill children at time of exposure to early or late parenteral nutrition in determining the impact hereof on long-term neurocognitive development:**

**a secondary analysis of the PEPaNIC-RCT**

Ines Verlinden, M.D.^1*^, Karolijn Dulfer, Ph.D.^2*^, Ilse Vanhorebeek, Ph.D.^1^, Fabian Güiza, Ph.D.^1^, José A. Hordijk M. Psych.^2^, Pieter J Wouters, M. Sc.^1^, Gonzalo Garcia Guerra, M.D.^3^, Koen F. Joosten, M.D., Ph.D.^2^, Sascha C. Verbruggen, M.D., Ph.D.^2^, Greet Van den Berghe, M.D., Ph.D.^1^

^1^Clinical Division and Laboratory of Intensive Care Medicine, Department of Cellular and Molecular Medicine, KU Leuven, Leuven, Belgium; ^2^Intensive Care Unit, Department of Paediatrics and Paediatric Surgery, Erasmus Medical Centre, Sophia Children’s Hospital, Rotterdam, The Netherlands; ^3^Department of Paediatrics, Intensive Care Unit, University of Alberta, Stollery Children's Hospital, Edmonton, Canada. *contributed equally.

**Address correspondence to:** Greet Van den Berghe, Clinical Division and Laboratory of Intensive Care Medicine, KU Leuven, Herestraat 49, B-3000 Leuven, Belgium. Phone: 32-16-34-40-21; Fax: 32-16-34-40-15; E-mail: greet.vandenberghe@kuleuven.be; ORCID: 0000-0002-5320-1362

SUPPLEMENTARY APPENDIX METHODS

**Methods S1. Definition of ‘syndrome’**

**Methods S2. Definition of educational and occupational level of parents**

**Methods S3. Detailed description of outcome measures**

SUPPLEMENTARY APPENDIX TABLES

**Table S1. Macronutrient and caloric target per centre**

**Table S2. Average total macronutrient doses administered up to each of the first 7 days in PICU, expressed as percentages of the reference doses for age and weight**

SUPPLEMENTARY APPENDIX FIGURES

**Figure S1. Total energy intake of the early-PN and late-PN patients of each age group for the first 7 days in PICU**

**Figure S2. Blood glucose levels of the early-PN and late-PN patients of each age group for the first 7 days in PICU**

REFERENCES

SUPPLEMENTARY APPENDIX METHODS

**Methods S1. Definition of ‘syndrome’**

A prerandomisation syndrome or illness *a priori* defined as affecting or possibly affecting neurocognitive development, and which is subdivided in the following categories:

- Genetically confirmed syndrome or pathogenic chromosomal abnormality
- Clearly defined syndrome, association or malformation without (identified) genetic aberration
- Polymalformative syndrome of unknown aetiology
- Clear auditory or visual impairment without specified syndrome
- Congenital hypothyroidism due to thyroid agenesis
- Brain tumour or tumour with intracranial metastatic disease
- Paedopsychiatric disorder (e.g. autism spectrum disorder, (treatment for) attention deficit hyperactivity disorder)
- Severe medical disorder, not primarily neurologic, but suspected to alter psychomotor and/or mental performance
- Severe neonatal problem (e.g. severe asphyxia)
- Severe craniocerebral trauma or near-drowning
- Severe infectious encephalitis or drug-induced encephalopathy
- Infectious meningitis, encephalitis or Guillain-Barré
- Resuscitation and/or need for extracorporeal membrane oxygenation prior to randomisation
- Severe convulsions or stroke prior to randomisation

**Methods S2. Definition of educational and occupational level of parents**

**Educational level of parents**

The educational level is calculated based upon the 3-point scale subdivision as made by the Algemene Directie Statistiek (Belgium; statbel.fgov.be/nl/) and the Centraal Bureau voor de Statistiek (The Netherlands; statline.cbs.nl): low (1), middle (2) and high (3) educational level. The average score of the paternal and maternal educational level was calculated. Categories where than made as followed: average 1 or 1·5 = category 1, average 2 or 2·5 = category 2, average 3 = category 3.

**Occupational level of parents**

The occupational level is calculated based upon the international Isco System 4-point scale for professions.^1^ In case one of the parents filled in two jobs in the questionnaire, the highest Isco code level was used. In case ‘unemployed’, ‘disabled’, ‘student’, or ‘housewife/houseman’ was filled in, an Isco code level of 1 was given to that parent. When the parents described their profession as ‘employee’, ‘worker’, ‘liberal profession’, or ‘retired’, they were given an Isco code level of 2. The average score of the paternal and maternal occupational level was calculated. Categories where than made as followed: average 1 or 1·5 = category 1, average 2 or 2·5 = category 2, average 3 or 3·5 = category 3, average 4 = category 4.

**Methods S3. Detailed description of outcome measures**

**Medical assessment**

*Anthropometric data*

At the beginning of the follow-up visit, height (in cm), body weight (in kg) and head circumference (in cm) were measured.

*Health status*

In an interview with the parents, the need for medical support of all kind during the past two years for healthy control children and during the 2 years following the index PICU admission for patients, was recorded. The hospital admissions because of surgery or a medical reason, and the occurrence of a psychiatric diagnosis were documented.

*Clinical neurological examination*

In order to assess whether there were gross neurological abnormalities, during a structured clinical neurological examination, signs of major neurologic dysfunction were detected in the following domains: interaction/language skills, gross motor function, involuntary movements, reflexes, coordination and balance, fine motor function, cranial nerves, and special senses (sensory, visual, and auditory function). These were all scored normal or abnormal. A normal result for each of these domains was given 1 point and the sum was made of all the abnormal results, with a range of 0-8.

**Neurocognitive testing**

*Patients/Parents-reported outcomes (PROs)*

Executive functioning was measured with the Behaviour Rating Inventory of Executive Function (BRIEF-P 2·5-5 years, BRIEF 6-18 years), filled out by the parents/caregivers of the child. Overlapping scales and indices of both questionnaires (inhibition, flexibility, emotional control, working memory, panning and organisation, metacognition) and a total score were analysed (T-scores, with mean 50 and SD 10). Inhibitory control refers to the ability to withhold initial responses in contexts where they are not appropriate and emotional control to respond on ongoing experiences with a range of emotions that is socially tolerable and sufficiently flexible to permit spontaneous reactions. Cognitive flexibility is the ability to shift attentional focus between tasks and mental sets. Emotional control is the ability to manage and control your emotions in order to achieve a goal or complete a task. Panning and organisation involves managing current or future tasks by setting goals and establishing the steps needed to complete the task. Working memory is a cognitive system that temporarily maintains and manipulates information. Metacognition is the awareness and understanding of one’s own thought processes, ‘thinking about thinking’.^2-4^

Behavioural and emotional problems were assessed by the parents/caregivers of the child with the Child behaviour checklist (CBCL 1·5-5 years or CBCL 6-18 years). Internalising, externalising and total problems were analysed. Internalising problems refers to anxious, depressed, withdrawn and over-controlled behaviour and externalising problems to aggressive, hyperactive, noncompliant, and under controlled behaviour. In the total score for the behavioural and emotional problems, not only internalising and externalising problems, but also sleep abnormalities for younger children and social, thinking and attention abnormalities for older children are included.^5-6^

*Intelligence*

General intellectual ability was assessed with use of age-appropriate versions of the Wechsler Intelligence Quotient (IQ) tests. The Wechsler Preschool and Primary Scale of Intelligence (WPPSI-III-NL^7^) was used for children aged 2·5 years - 5 years 11 months (one version for age range 2 years 6 months - 3 years 11 months, and another version for age range 4 years - 5 years 11 months), the Wechsler Intelligence Scale for Children (WISC-II-NL^8^) was used for children aged 6 years - 16 years 11 months, and the Wechsler Adult Intelligence scale (WAIS-IV-NL^9^) for adolescents who were 17 years or older. For all these testes Total IQ, Verbal IQ and Performal IQ scores (test-mean 100, SD 15) were computed.

*Visual-motor integration*

We used the beery Developmental Test of visual-motor integration 6th Edition to assess the ability to integrate visual and motor functions (total scaled score with test-mean 10, SD 3). This involves eye-hand coordination.^10^

SUPPLEMENTARY APPENDIX TABLES

**Table S1. Macronutrient and caloric target per centre**^11^

| **Centre** | **First day** | **Subsequent stay** |
| --- | --- | --- |
| Leuven, Belgium | First 10 kg: 100 kcal/kg  10-20 kg: + 50 kcal/kg  >20 kg: + 20 kcal/kg  (adjusted downward when fluid restriction required) | |
| Rotterdam, The Netherlands | EN: basal metabolic rate by Schofield-weight^12^ PN: ESPGHAN^13^ | EN: Recommended Dietary Allowances^14^ PN: ESPGHAN^13^ |
| Edmonton, Canada | Resting energy expenditure by indirect calorimetry. If indirect calorimetry impossible: 65% of basal metabolic rate (FAOWHO^15^) | Adjusted daily by the dietitian based on clinical information |

EN=enteral nutrition, PN=parenteral nutrition.

**Table S2. Average total macronutrient doses administered up to each of the first 7 days in PICU, expressed as percentages of the reference doses for age and weight**

|  | **Glucose** | | **Amino acids** | | **Lipids** | |
| --- | --- | --- | --- | --- | --- | --- |
| **Dose up to day** ^a^ | **Early PN** | **Late PN** | **Early PN** | **Late PN** | **Early PN** | **Late PN** |
| 1 | 60.3 (37.1-81.7) | 16.5 (11.7-24.2) | 74.0 (0.0-102.2) | 0.0 (0.0-0.0) | 0.0 (0.0-0.0) | 0.0 (0.0-0.0) |
| 2 | 70.4 (47.7-86.3) | 19.1 (14.3-32.6) | 87.4 (45.8-109.2) | 0.0 (0.0-6.6) | 15.9 (4.6-49.1) | 0.0 (0.0-11.2) |
| 3 | 61.9 (43.1-75.7) | 19.9 (14.4-31.8) | 80.0 (56.7-99.4) | 2.0 (0.0-13.2) | 36.6 (21.7-73.7) | 3.5 (0.0-28.2) |
| 4 | 60.1 (42.1-72.1) | 20.1 (14.4-31.9) | 75.7 (59.1-89.7) | 3.2 (0.0-19.5) | 49.5 (34.2-79.1) | 6.8 (0.0-41.2) |
| 5 | 59.2 (42.3-70.9) | 20.7 (14.8-31.8) | 74.1 (59.2-87.9) | 4.7 (0.0-27.3) | 60.9 (46.2-93.9) | 9.6 (0.0-46.8) |
| 6 | 56.1 (40.8-70.9) | 23.7 (15.6-34.5) | 75.0 (57.5-85.5) | 6.8 (0.5-33.0) | 67.9 (51.3-98.7) | 14.6 (1.4-54.4) |
| 7 | 56.8 (41.6-69.5) | 24.4 (15.3-36.0) | 73.8 (60.1-86.8) | 11.7 (1.7-34.3) | 73.7 (57.2-106.0) | 23.7 (2.3-60.5) |

^a^ Average daily doses of the 3 macronutrient classes administered up to each of the first seven days in PICU are expressed as percentages of the reference doses for age/weight as described in nutritional guidelines summarised in Table S1.^11^ Data represent medians and interquartile ranges.

SUPPLEMENTARY APPENDIX FIGURES

**Figure S1. Total energy intake of the early-PN and late-PN patients of each age group for the first 7 days in PICU**

**
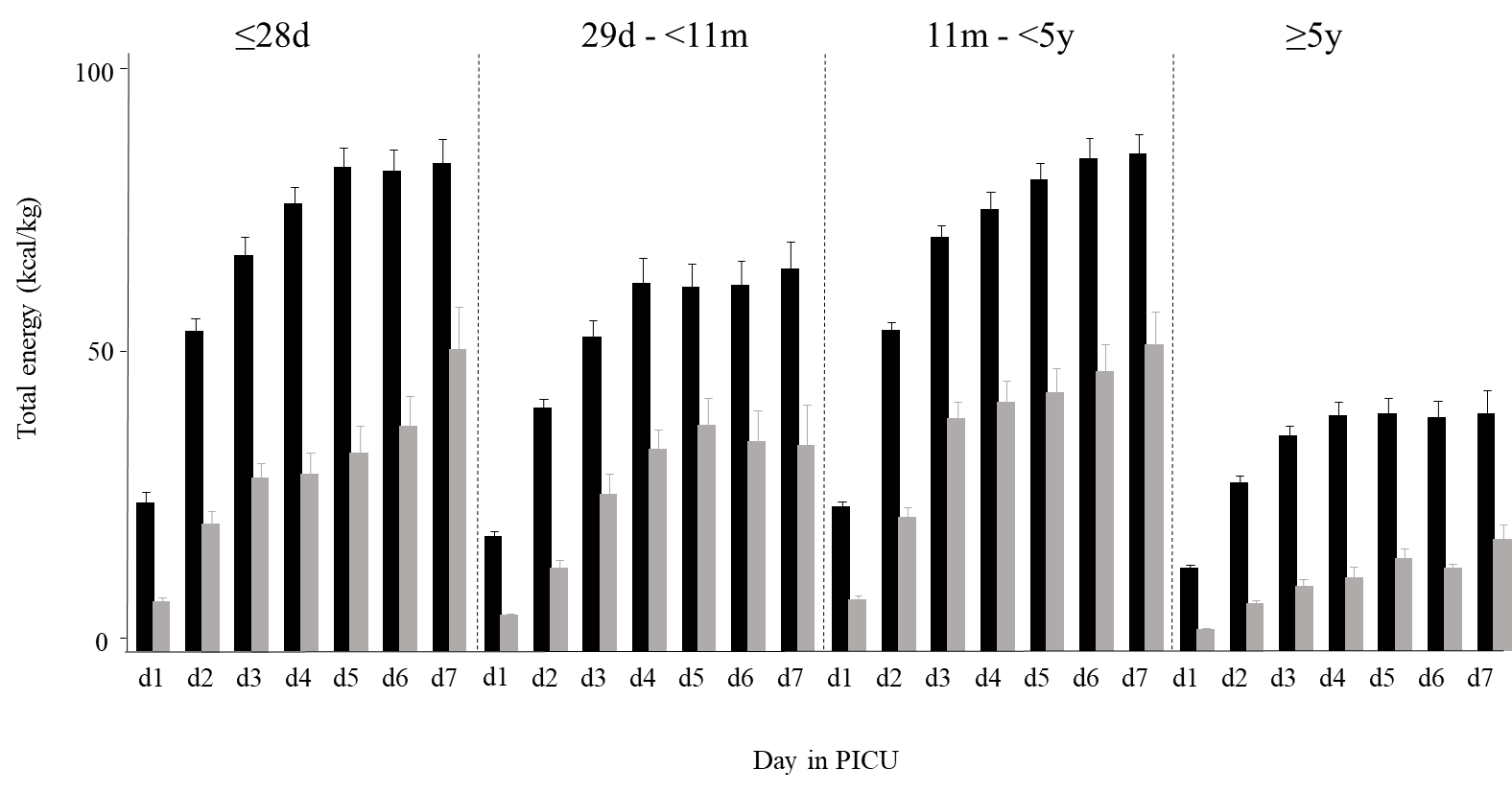
**

Black bars represent the daily total amount of energy (kilocalories per kg) for early-PN patients and grey bars for late-PN patients. Data are presented as means and standard errors.

**Figure S2. Blood glucose levels of the early-PN and late-PN patients of each age group for the first 7 days in PICU**

**
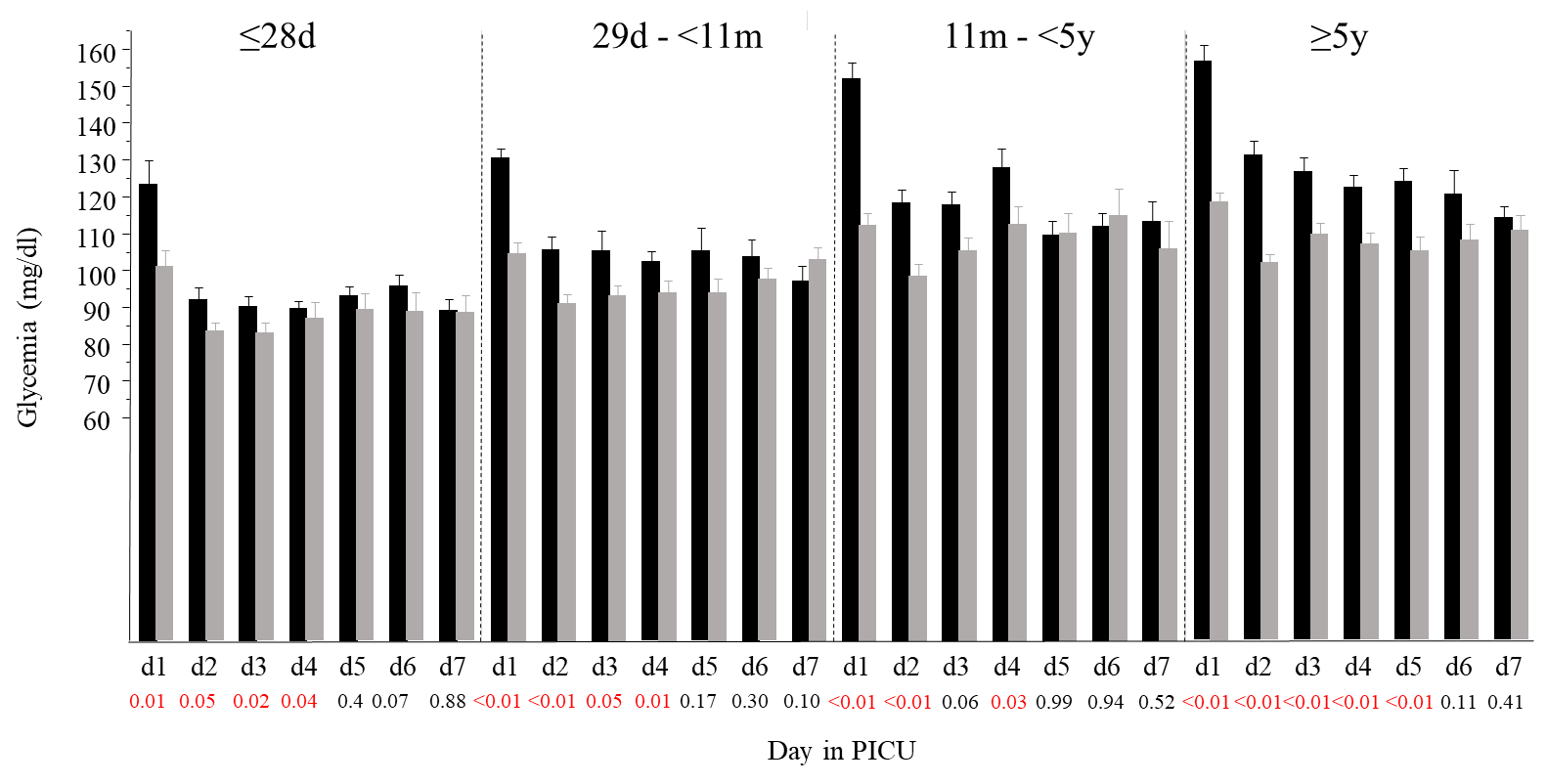
**

Black bars represent the average glycaemia (milligram per decilitre) per day for early-PN patients and grey bars for late-PN patients in each age group. Data are presented as means and standard errors.

REFERENCES

1. https://www.ilo.org/public/english/bureau/stat/isco/isco08/index.htm
2. Mesotten D, Gielen M, Sterken C, et al. Neurocognitive development of children 4 years after critical illness and treatment with tight glucose control: a randomized controlled trial. *JAMA* 2012; **308:** 1641-50.
3. Van der Heijden KB, Suurland J, De Sonneville LM, et al. BRIEF-P Vragenlijst voor executieve functies voor 2- tot 5-jarigen: Handleiding. Amsterdam: Hogrefe, 2013.
4. Huizinga M, Smidts D. BRIEF Vragenlijst executieve functies voor 5- tot 18-jarigen: Handleiding. Amsterdam: Hogrefe, 2012.
5. Achenbach TM, Rescorla LA. Manual for the ASEBA Preschool Forms and Profiles. Burlington: University of Vermond, Research Center for Children, Youth, and Families, 2000.
6. Verhulst FC, Van der Ende J. Handleiding ASEBA. Vragenlijsten voor leeftijden 6 tot en met 18 jaar [ASEBA Manual Questionnaires for ages 6 to 18 years]. Rotterdam: ASEBA Nederland, 2013.
7. Hendriksen J, Hurks P. WPPSI-III-NL. Wechsler Preschool and Primary Scale of Intelligence: Handleiding. Amsterdam: Pearson, 2010.
8. Wechsler D. WISC-III Nederlandstalige bewerking. Handleiding. Amsterdam: Pearson, 2005.
9. Wechsler D. WAIS-III Nederlandstalige bewerking. Afname en Scoringshandleiding. Amsterdam: Pearson, 2012.
10. Beery KE, Buktenica NA, Beery NA. The Beery-Buktenica Developmental Test of Visual-Motor Integration, 6th edn (BEERYTM VMI). Amsterdam: Pearson, 2010.
11. Fivez T, Kerklaan D, Mesotten D, et al. Early versus Late Parenteral Nutrition in Critically Ill Children. *N Engl J Med* 2016; **374:** 1111-22.
12. Schofield WN, Predicting basal metabolic rate, new standards and review of previous work. *Hum Nutr Clin Nutr* 1985; **39**: S5-41.
13. Koletzko B, Goulet O, Hunt J, et al. Guidelines on Paediatric Parenteral Nutrition of the

European Society of Paediatric Gastroenterology, Hepatology and Nutrition (ESPGHAN) and the European Society for Clinical Nutrition and Metabolism (ESPEN), Supported by the European Society of Paediatric Research (ESPR). *J Pediatr Gastroenterol Nutr* 2005; **41**: S1-87.

1. Dietary Reference Intake: energy, protein and digestible carbohydrates. Health Council of the Netherlands: The Hague 2001.
2. Energy and protein requirements. Report of a joint FAO/WHO/UNU Expert Consultation. *World Health Organ Tech Rep Ser* 1985; **724**: 1-206.
